# Prevalence of sexually transmissible infections in adolescents treated in a family planning outpatient clinic for adolescents in the western Amazon

**DOI:** 10.1371/journal.pone.0287633

**Published:** 2023-06-23

**Authors:** Ida Peréa Monteiro, Camila Flávia Gomes Azzi, João Paolo Bilibio, Pedro Sadi Monteiro, Giordana Campos Braga, Nadjar Nitz

**Affiliations:** 1 Municipal Health Department, Mãe Esperança Municipal Maternity, Porto Velho, Rondônia, Brazil; 2 Molecular Biology Laboratory, Central Laboratory of Public Health of Rondônia, Porto Velho, Rondônia, Brazil; 3 Faculty of Medicine, Centro Universitário de Brusque–UNIFEBE, Brusque, Santa Catarina, Brazil; 4 Department of Nursing, University of Brasilia, Brasília, Brazil; 5 Department of Social Medicine, Ribeirao Preto Medical School, University of São Paulo, Ribeirao Preto, São Paulo, Brazil; 6 Faculty of Medicine, Interdisciplinary Laboratory of Biosciences, University of Brasília, Brasília, Brazil; PhD, PLOS, UNITED KINGDOM

## Abstract

Sexually transmitted infections (STIs) are among the most common public health problems worldwide, especially among adolescents and young adults, who account for almost 50% of all STI patients. Studies on the subject in the western Amazon are limited. This study aimed to evaluate the prevalence of STIs (chlamydia, gonorrhea, trichomoniasis, herpes simplex virus, syphilis, human immunodeficiency virus [HIV], hepatitis B, and hepatitis C) in adolescents treated at a family planning outpatient clinic in the western Amazon: Porto Velho, Rondônia, Brazil. A total of 196 adolescents were enrolled. During the gynecological examination, endocervical samples were collected to test for four STIs (chlamydia, gonorrhea, trichomoniasis, and herpes simplex virus), and blood samples were collected for the detection of HIV, syphilis, and hepatitis B and C. The mean age was 17.3 ± 1.5 years, the age at sexarche was 14.4 ± 1.6 years, and 54.6% of participants had their first sexual intercourse at 14 years or younger. Only 1.0% of the adolescents used condoms in all sexual relations, and 19.9% had casual partner(s) in the last year. In the evaluation of prevalence, we found that 32% of the adolescents had at least one STI, with the most prevalent being chlamydia (23%), followed by trichomoniasis (5.6%), herpes simplex (4.6%), and gonorrhea (3.1%). No positive cases of hepatitis B, hepatitis C, or HIV were detected, but 1% of the adolescents tested positive for syphilis. These indicators will support more effective health care strategies aimed at improving the quality of life of populations in this region of the western Amazon. In conclusion, our findings demonstrated high rates of STIs in the studied patients, reinforcing the need to expand epidemiological studies to implement more appropriate public policies and intervention strategies to prevent STIs in adolescents and other vulnerable populations in the western Amazon.

## Introduction

Sexually transmitted infections (STIs) remain a serious public health problem worldwide. The yearly incidence estimated by the World Health Organization (WHO) is approximately 376 million new infections with the four curable STIs (chlamydia, gonorrhea, trichomoniasis, and syphilis), which represents an average of more than one million new infections per day; these STIs are most common in women [1]. These infections are also implicated in the increased risk of infection by human immunodeficiency virus (HIV). In addition, people with STIs often suffer stigma, stereotypes, vulnerability, shame, and sexual violence [2].

Women have the highest prevalence of STIs [1], and another group of great vulnerability is adolescents, both behaviorally and biologically. From the behavioral point of view, adolescents are more likely to engage in high-risk sexual behaviors, such as sex without a condom or simultaneous partners, because their prefrontal cortex, which is responsible for executive function, is still developing [3]. From the biological point of view, female adolescents are particularly susceptible to STIs due to their lower production of cervical mucus and greater cervical ectopy [4,5]. Therefore, female adolescents are more likely than adults to experience infections, making them one of the groups most susceptible to STIs.

Adolescents are also the group most likely to have long-term health problems due to STIs if left untreated. Chlamydia, gonorrhea, and trichomoniasis may cause infertility and adverse outcomes at delivery [5,6]. Cervical, oropharyngeal, and rectal cancer are associated with certain human papilloma virus strains [7]. Syphilis can cause long-term complications, such as damage to the nervous system and cardiovascular system, and congenital syphilis can cause serious complications leading to infant death. STIs have been associated with increased rates of contamination by hepatitis B, hepatitis C, and HIV [8]. Although their high prevalence and high morbidity are well known, there are relatively limited data on STIs in adolescents.

Knowing the STI prevalence is crucial for the implementation of public health policies and more effective preventive measures. The WHO has shown the importance of knowing the prevalence of STIs to increase the effectiveness public measures regarding prevention and treatment and to break chains of transmission [1]. Accordingly, the World Health Assembly adopted the 2016–2021 Global Health Sector Strategy on Sexually Transmitted Infections [9]. This strategy includes the rapid expansion of interventions and evidence-based services to end STIs as public health problems by 2030. However, the limited research on the prevalence of STIs and unsatisfactory screening programs complicate the implementation of control strategies in Brazil.

Although knowing the prevalence of STIs in adolescents is essential for adequate planning for the control of these diseases, the global estimates are based on a relatively small number of surveys [1,10]. Some STIs are found more commonly among adolescents and young people than others, and in most studies, girls appear to be more frequently affected than boys [1,10–12]. In several studies, adolescent girls accounted for the highest level of chlamydial infection detected by culture among all age groups, and the prevalence of chlamydial infection ranged from less than 10% to more than 40% depending on the assessed group and associated risks [1,10,11,13–15]. Overall, data on gonorrhea among adolescents are still very limited. Existing studies show that the prevalence of gonorrhea among adolescent girls is usually lower than that of chlamydia [1,10,11,13,15]. In the Americas, there is an estimated incidence of 29.8 million cases of chlamydia, 13.8 million cases of gonorrhea, and 2 million cases of syphilis [1].

One STI prevalence study conducted by the Brazilian Ministry of Health covered only six Brazilian capitals, including two cities in the Southeast Region and one city each in the other regions [16]. The research demonstrated high prevalence rates of syphilis among pregnant women (> 1%) and STIs among adolescents (> 10%) but failed to adequately cover all of Brazil, especially the North Region [16]. Studies in the western Amazon region are scarce and would be of fundamental importance for public policy planning. In Rondônia, one of the 27 federal units of Brazil, located in the western Amazon and bordering Bolivia, there has been no study on the prevalence of STIs.

Due to the importance of adequate public measures for prevention and treatment, in addition to the scarcity of studies on STIs in the western Amazon region of Brazil, especially in adolescents, the present study aimed to determine the prevalence of the pathogens responsible for the most common STIs, including *Chlamydia trachomatis* (chlamydia), *Neisseria gonorrhoeae* (gonorrhea), *Trichomonas vaginalis* (trichomoniasis), Treponema pallidum subspecies *pallidum* (syphilis), HIV, hepatitis B, hepatitis C, and herpes simplex viruses 1 and 2, in adolescents attending a family planning outpatient clinic in Porto Velho city, Rondônia State, Brazil.

## Materials and methods

This cross-sectional epidemiological study was conducted with female adolescents treated at the Rafael Vaz e Silva Family Planning Outpatient Clinic between November 2017 and March 2019. Written informed consent, duly approved by the national research ethics committee, was obtained from all participants. Those of legal age received the term in writing, read and signed the consent form. Adolescents under 18 years of age read and signed the Free and Informed Consent Form together with their parents or legal guardian to participate in the study. The study was approved by the Research Ethics Committee of the Universidade Federal de Rondônia (Federal University of Rondônia), Porto Velho, Brazil (protocol number 1.701763).

### Study population

All adolescents seen at the family planning clinic were invited to participate in the study. This clinic is a resource for adolescents who wish to plan their contraception. A total of 196 adolescents aged 14–19 years were selected for the study. Patients with a confirmed diagnosis of any of the STIs investigated in this study were excluded for that specific pathology but were included in the screening for the other STIs. Patients who had used antibiotic therapy or any intravaginal chemical substance in the last two weeks preceding the day of the examination were excluded, as were indigenous and foreign adolescents who were unable to communicate. All patients underwent anamnesis, general physical and gynecological examinations, and sample collection. A questionnaire was administered to assess the epidemiological-sociodemographic profiles of the patients. All adolescents and their guardians were informed of the results of the tests performed and were instructed to undergo appropriate treatment if necessary.

Endocervical samples were collected during the gynecological examination to be tested for four STIs (*C*. *trachomatis*, *N*. *gonorrhoeae*, *T*. *vaginalis*, herpes simplex). After this step, in the last stage of care, blood samples were collected by finger prick with a retractable lancet, and blood was drawn with a sterile capillary tube; this sample was used for the detection of HIV, syphilis, and hepatitis B and C.

### STI detection of sexually transmitted pathogens by multiplex PCR

The samples that were collected from the endocervix with a cytobrush were transferred to Eppendorf tubes (Eppendorf, Hamburg, Germany) containing 1.8 mL of sterile saline solution. Genomic DNA was extracted with Biopur Mini Spin Plus (Mobius Life Science, Paraná, Brazil) following the manufacturer’s instructions. DNA was quantified in a NanoDrop 2000 spectrophotometer (Thermo Scientific, Epsom, UK). TaqMan multiplex real-time polymerase chain reaction (qRT‒PCR) was performed using the RUO Multiplex Sexually Transmitted Diseases 9 FTD kit (Fast Track Diagnostics, Junglinster, Luxembourg) for the detection of *C*. *trachomatis*, *N*. *gonorrhoeae*, *T*. *vaginalis*, and herpes simplex viruses 1 and 2. The reactions were performed according to the manufacturer’s instructions using the 7500 Fast Real-Time PCR system (Applied Biosystems, MA, USA) as previously described [17]. The total volume used for the assay was 25 μL for each dilution, with 15 μL of amplification mixture and 10 μL of the sample. The thermocycling program was 50°C for 15 minutes for reverse transcription, followed by activation of PCR at 94°C for 1 minute, then 40 cycles of 8 seconds at 94°C and 1 minute at 60°C. Amplification was carried out on 96-well plates (Optical 96-Well Reaction Plate, MicroAmp®) using the thermocycler 7500 Real-time PCR System (Applied Biosystems, CA, USA). The molecular procedures were performed at the Laboratory of Molecular Biology of the Central Laboratory of Public Health of Rondônia.

### Immunological rapid detection tests for sexually transmitted pathogens

Blood samples were collected using the digital pulse technique with the aid of a retractable lancet into sterile capillary tubes. The samples were dispensed on a specific slide for each test for the detection of HIV, syphilis, and hepatitis B and C. For HIV detection, we used the HIV 1/2/O Tri-line Human Immunodeficiency Virus Rapid Test (ABON Biopharm, Zhejiang, China). For syphilis detection, we used the Immuno-Rapid Syphilis kit (Wama Diagnóstica, Sao Paulo, Brazil), hepatitis B was detected with the rapid immunochromatographic VIKIA^®^ HBs Ag test (bioMérieux, Marcy-l’Étoile, France), and for hepatitis C detection, we used the Alere HCV Kit (Alere, Massachusetts, USA). The protocol established by each manufacturer was carefully followed.

### Statistical analysis

The sample size was calculated before study initiation using the program WinPepi version 11.65. Due to the few studies on the prevalence of STIs in Brazil, with especially few studies on female adolescents, the sample size calculation was based on the study carried out by the Brazilian Ministry of Health on the prevalence and relative frequencies of sexually transmitted diseases (STDs) in selected populations of six Brazilian capitals [16]. To this end, the prevalence of syphilis, gonorrhea and chlamydia was used in pregnant women in six Brazilian capitals, which included Manaus, located in the northern region of Brazil, in the western Amazon. Therefore, based on the prevalence of syphilis (average of 2.6% and maximum of 4.4%), the required sample size was 51 (if the rate was 26 per 1000, with confidence level = 95% and acceptable diff. = 44 per 1000). Based on the prevalence of gonorrhea (average 1.5% and maximum 4.3%), the required sample size was 73 (if the rate is 15 per 1000, confidence level = 95%. and acceptable diff. = 28 per 1000), and based on the prevalence of chlamydia (average of 9.4% and maximum of 14.8%), the required sample size was 113 (if the rate is 94 per 1000 with a confidence level = 95% and acceptable diff. = 54 per 1000). Then, based on the need for the largest n, estimating a C.I. of up to 108 per 1000, the final required sample size was at least 189.

The data were entered into and analyzed using SPSS v.25 (IBM Corp., New York, USA) and EPI-INFO 7.2 (Centers for Disease 168 Control and Prevention, GeorgiaGA, USA) for Windows. The results were reported using the mean, median, minimum, maximum, standard deviation, and absolute and relative frequencies (percentages) for the quantitative variables. To compare the qualitative variables, the chi-squared test or Fisher’s exact test was performed. Statistical significance was established as P < 0.05.

## Results

**Fig 1:**
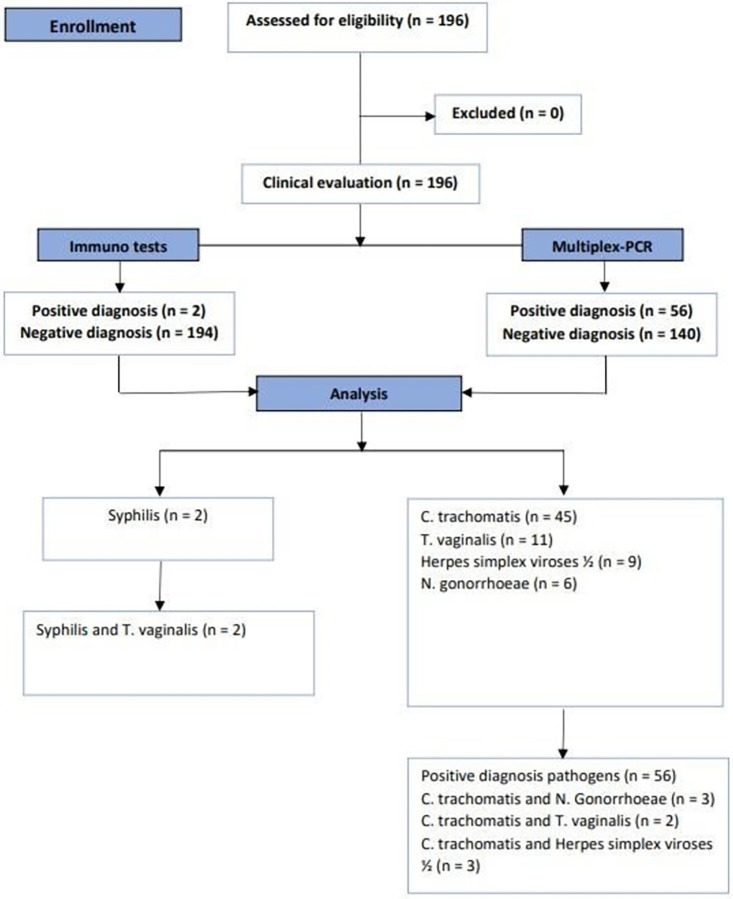
Flowchart representing the study design and the results of the sexually transmitted infections detected in adolescents treated in the family planning outpatient clinic.

In total, 196 adolescents aged 14–19 years were included in the study. **Fig 1** shows a flowchart of the study with details of the number of STIs detected in adolescents.

The epidemiological-sociodemographic profile of the adolescents treated at the family planning outpatient clinic is shown in **Table 1**. The mean age of the adolescents was 17.3 ± 1.5 years, and the age at sexarche ranged from 8 to 18 years (14.4 ± 1.6). A total of 52.6% of the adolescents said they were homemakers, and 45.9% had only 1 to 8 years of schooling.

**Table 1:** Epidemiological-sociodemographic profiles of adolescents treated at a family planning outpatient clinic in Porto Velho city, Rondônia State, Brazil (n = 196).

	Mean	SD	Median (min-max)
**Age (years)**	17.3	1.5	17 (14–19)
	**N**	**%**	**Cumulative %**
**14–15 years**	30	15.3%	15.3%
**16–17 years**	69	35.2%	50.5%
**18–19 years**	97	49.5%	100%
**Occupation**	**N**	**%**	
**Homemaker**	103	52.6%	
**Student**	82	41.8%	
**Merchant**	6	3.0%	
**Self-employed**	5	2.6%	
**Years of schooling**	**N**	**%**	**Cumulative %**
**1–8 years**	90	45.9%	45.8%
**9–12**	101	51.5%	97.3%
**> 12**	5	2.6%	100%
**Ethnicity**	**N**	**%**	
**White**	22	11.2%	
**Black**	20	10.2%	
**Biracial**	154	78.6%	
**Age at menarche**	**Mean**	**SD**	**Median (min-max)**
	12.2	1.4	12 (9–16)

None of the patients reported any genital symptoms at the time of care. During the anamnesis, the patient was asked whether she had ever had any genital symptoms in the past. The most frequent was vaginal discharge (65.8%), followed by small blisters or vesicles (9.2%), wounds (8.2%), and genital warts (5.1%). The sexual profile and marital status of the adolescents treated at the family planning outpatient clinic are shown in **Table 2**. The mean age at sexarche was 14.4 years, and 54.6% had their first sexual intercourse at 14 years of age or younger. Only 1.0% of the adolescents used condoms in all sexual relations, 60.7% had more than one partner in their lifetime, and 19.9% had casual partners in the past year. In terms of the number of pregnancies, 86.3% had one or more pregnancies.

**Table 2:** Sexual profiles and marital statuses of adolescents treated at a family planning outpatient clinic in Porto Velho city, Rondônia State, Brazil (n = 196).

	Mean	SD	Median (min-max)
**Age at sexarche**	14.4	1.6	14 (8–18)
	**N**	**%**	**Cumulative %**
**8 years**	2	1.0%	1.0%
**9 years**	0	0.0%	1.0%
**10 years**	1	0.5%	1.5%
**11 years**	2	1.0%	2.5%
**12 years**	10	5.1%	7.6%
**13 years**	37	18.9%	26.5%
**14 years**	55	28.1%	54.6%
**15 years**	47	24.0%	79.4%
**16 years**	27	13.8%	93.2%
**17 years**	11	5.6%	98.8%
**18 years**	4	2.0%	100%
**Have you had > 1 sexual partner in your life?**	**No**	**Yes**	**No answer**
**N (%)**	76 (38.8%)	119 (60.7%)	1 (0.5%)
**Did you use condoms in all sexual relations?**	**No**	**Yes**	**No answer**
**N (%)**	175 (89.3%)	2 (1.0%)	19 (9.7%)
**Have you had relationships with casual partners in the past 12 months?**	**No**	**Yes**	**No answer**
**N (%)**	156 (79.6%)	39 (19.9%)	1 (0.5%)
**Number of pregnancies you have had?**	**N**	**%**	**Cumulative %**
**3 or more**	5	2.6%	2.6%
**2**	35	17.9%	20.5%
**1**	129	65.8%	86.3%
**0**	27	13.8%	100%
**What is your marital status?**	**N**	**%**	
**Single**	55	28.1%	
**Stable relationship**	141	71.9%	

The prevalence of *C*. *trachomatis*, *T*. *vaginalis*, herpes simplex, and *N*. *gonorrhoeae* in the endocervix samples of adolescents treated at the family planning outpatient clinic is shown in **Table 3**. The most prevalent STI among the adolescents evaluated was *C*. *trachomatis* (23%); *T*. *vaginalis*, herpes simplex, and *N*. *gonorrhoeae* had prevalence rates of 5.6%, 4.6%, and 3.1%, respectively.

**Table 3:** Prevalence rates of *C*. *trachomatis*, *T*. *vaginalis*, herpes simplex, and *N*. *gonorrhoeae* in endocervix samples from the adolescents treated at a family planning outpatient clinic in Porto Velho city, Rondônia State, Brazil (n = 196).

Sexually transmitted infection	Positive cases	Prevalence
*Chlamydia trachomatis*	45	23.0%
*Trichomonas vaginalis*	11	5.6%
Herpes simplex viruses 1 and 2	9	4.6%
*Neisseria gonorrhoeae*	6	3.1%

The prevalence of syphilis, hepatitis B, hepatitis C, and HIV from the rapid testing of blood samples was determined: 2 (1.02%) adolescents tested positive for syphilis, and there were no positive cases of hepatitis B, hepatitis C, or HIV.

In addition, 5% of the patients (10/196) were positive for concomitant STIs. For instance, 1% of the patients (2/196) had syphilis and trichomoniasis or *C*. *trachomatis* and *T*. *vaginalis* infections, while 1.5% of the patients (3/196) were coinfected with *C*. *trachomatis* and *N*. *gonorrhoeae* or *C*. *trachomatis* and herpes simplex viruses 1/2. We found that 32% of the adolescents had at least one STI infection.

To assess some types of behavior by adolescents that could influence the prevalence of STIs, we made some associations. Among these, we evaluated the association between early sexarche and the risk of STI (**Table 4**). We found that sexarche before 14 years of age increased the risk of contracting a *T*. *vaginalis* infection with OR 1.5 (CI 1.1–2.1, P = 0.039) and the risk of contracting *N*. *gonorrhoeae* with OR 1.9 (1.3–2.7, P = 0.029). We found no association with the other STIs evaluated (*C*. *trachomati*s and herpes simplex viruses 1 and 2).

**Table 4:** Associations between age at sexarche (< 14 years or > 14 years) and prevalence of STIs %.

	Sexarche < 14 years	Sexarche >14 years	OR (IC 95%)	P
***Chlamydia trachomatis***	**% (n)**	**% (n)**		
Positive	60.0% (27)	40.0% (18)	1.1 (0.8–1.5)	0.406^a^
Negative	53.0% (80)	47.0 (71)		
***Trichomonas vaginalis***	**% (n)**	**% (n)**		
Positive	83.3% (10)	16.7% (2)	1.5 (1.1–2.1)	0.039^b^
Negative	52.7% (97)	47.3 (87)		
**Herpes simplex viruses 1 and 2**	**% (n)**	**% (n)**		
Positive	55.6% (5)	44.4% (4)	1.0 (0.5–1.8)	0.953^b^
Negative	54.5% (102)	45.5% (85)		
***Neisseria gonorrhoeae***	**% (n)**	**% (n)**		
Positive	85.7% (6)	14.3% (1)	1.9 (1.3–2.7)	0.029^b^
Negative	43.9% (83)	56.1% (106)		

^**a**^ Chi-squared test or.

^**b**^ Fisher’s exact test.

We also evaluated other sociodemographic variables and the noted prevalence of STIs (having more than one sexual partner, casual partners in the past 12 months, marital status) (**Table 5)**. We found no association between any of these factors and STIs.

**Table 5:** Associations between socio demographic variables and prevalence of STIs.

	> 1 sexual partner in your life?		P	Casual partners in the past 12 months?		P ^a^	Marital status?		P
	**No**	**Yes**		**No**	**Yes**		**Single**	**Stable relationship**	
***Chlamydia trachomatis***	% (n)	% (n)		% (n)	% (n)		% (n)	% (n)	
Positive	35.6%(16)	64.4%(29)	0.614^**a**^	73.3%(33)	26.7%(12)	0.235^**a**^	20.0%(9)	80.0%(36)	0.170^**a**^
Negative	39.7%(60)	60.3%(91)		81.5%(123)	18.5%(28)		30.5%(46)	69.5%(105)	
***Trichomonas vaginalis***	% (n)	% (n)		% (n)	% (n)		% (n)	% (n)	
Positive	16.7%(2)	83.3%(10)	0.105^b^	83.3%(10)	16.7%(2)	0.740^b^	41.7%(5)	58.3%(7)	0.279^**a**^
Negative	40.2%(74)	59.8%(110)		79.3%(146)	20.7%(38)		27.2%(55)	72.8%(141)	
**Herpes simplex viruses 1 and 2**	% (n)	% (n)		% (n)	% (n)		% (n)	% (n)	
Positive	33.3%(73)	66.7%(114)	0.732^**a**^	88.9%(148)	11.1%(39)	0.479^**a**^	22.2%(2)	77.8%(134)	0.690^b^
Negative	39.0%(76)	61.0%(120)		79.1%(156)	20.95(40)		28.3%(53)	71.7%(141)	
***Neisseria gonorrhoeae***	% (n)	% (n)		% (n)	% (n)		% (n)	% (n)	
Positive	42.9%(3)	57.1%(116)	0.821^b^	57.1%(4)	42.9%(3)	0.133^b^	42.9%(52)	57.1%(4)	0.001^b^
Negative	38.6%(76)	61.4%(120)		80.4%(156)	19.6%(40)		27.5%(55)	72.5%(141)	

^**a**^ Chi-squared test.

^**b**^ Fisher’s exact test.

### Discussion

In this study, we evaluated the prevalence of the main STIs in female adolescents in the western Amazon population. The results of this study are of paramount importance for public health measures to prevent and treat STIs because no studies have been conducted in this region on the prevalence of STIs, especially in adolescents. We determined the prevalence of the four most common curable STIs (*C*. *trachomatis*, *N*. *gonorrhoeae*, *T*. *vaginalis*, and syphilis) and four viruses, HIV, hepatitis B and C, and herpes viruses 1 and 2, and found that 32% of the adolescents treated at the family planning outpatient clinic had at least one STI.

The most prevalent infectious agent was *C*. *trachomatis* at a rate of 23%, which is substantially higher than rates found in other regions of the Amazon, namely, 18% in the city of Belém, state of Pará based on two studies in the eastern Brazilian Amazon [18], 14.3% in Marajó Island, state of Pará [19], 18% in the city of Coari in the state of Amazonas based on two studies in the western Brazilian Amazon, and 15.1% in the city of Manaus in the state of Amazonas [16]. Even at such high prevalence rates, the association with risk variables are not easily identified; however, there is evidence linking prevalence to young age and sexual conduct, early sexarche [19–21], and low income in the Amazon region, which is inhabited by a largely rural population living in small communities with few public healthcare services or even basic programs focusing on reproductive health [19,22].

Precarious social conditions, a lack of economic opportunities, and risky sexual behavior are all closely associated with the incidence of STIs in young people [19,23]. The high prevalence of chlamydia found in our study is very worrisome, especially due to the potential risks associated with this disease. It is estimated that 80% of infected women are asymptomatic but may develop complications and sequelae in the long term, while others will have some symptoms involving urethritis, cervicitis, and pelvic inflammatory disease, the main cause of infertility, chronic pelvic pain, and ectopic pregnancy [13,24]. Other complications associated with chlamydia may come from maternal infection of the birth canal causing complications in the newborn, such as pneumonia and conjunctivitis [25]. Chlamydia infection also brings obstetric complications such as miscarriage, premature birth, low birth weight, fetal growth restriction, premature rupture of membranes, perinatal mortality, and endometritis [26].

Furthermore, our results also demonstrated a high prevalence of pathogens responsible for other curable STIs: *T*. *vaginalis* (5.6%) and *N*. *gonorrhoeae* (3.1%). Thus, we found that 32% of the adolescents evaluated had at least one STI. The WHO classifies a rate of 5% as an STI prevalence that is under control for a given population, while the rate in our study was six times higher than this limit. This result indicates that we are facing a serious public health problem in this community [9]. Comparing our rates with other published rates, adolescents from Pittsburgh, Pennsylvania, USA, also showed higher rates of *C*. *trachomatis* and *N*. *gonorrhoeae* at 16% and 12.5%, respectively [27]. Compared to other Brazilian findings, the prevalence of *N*. *gonorrhoeae* in our study (3.1%) was almost double that found by Britto et al. (1.6%) [28] and higher than that found by Jalil et al. (2.1%) [29]. A review by Kissinger et al. evaluated the worldwide prevalence of *T*. *vaginalis* infection and showed heterogeneity among different countries (25). In the USA, the prevalence rates were 2.3% among adolescents and 3.1% among adult women, while in Zimbabwe and Tanzania, the prevalence ranged from 9.5% to 11% among the two sexes. Belgium had the lowest prevalence among the countries included in the study at 0.37% [30].

The fact that we found higher rates of STIs among the adolescents in our study when compared, especially to other Brazilian regions, may be due to several factors. Analyzing the data in detail, we found that 54.6% of the girls in the study had their first sexual intercourse when they were 14 years old or younger. The age at sexarche is decreasing among girls [31]. This finding has important implications for the health of adolescent girls because of the association of young age at sexarche with subsequent health problems and subsequent risky sexual behavior. There are many factors associated with young age at sexarche, such as low socioeconomic status, limited education, divorced parents, living with a partner, not practicing a religion, smoking, and drug use [32–34].

Girls who have first sexual intercourse at 14 years of age or younger are less likely to use contraception such as condoms on this occasion, take longer before starting to use contraception in sexual intercourse, are more likely to have multiple sexual partners, have a higher risk of depression, have lower self-esteem and more episodes of regret, and have a higher risk of contracting STIs and cervical cancer [35]. Our data confirm this association between early sexarche and an increased risk of STIs. We found that early sexarche increases the risk of having a *T*. *vaginalis* infection by 1.5 times and by *N*. *gonorrhoeae* by 1.9 times; therefore, public measures that promote responsible and healthy sexuality should be provided in homes, schools, medical and community centers [36].

Another factor that is probably associated with the high prevalence of STIs found in our study is that almost 60% of adolescents had more than one sexual partner, and approximately 20% had casual partners in the last year. Early sexarche appears to have a significant correlation with multiple sexual partners in the last 12 months, which in turn may put these adolescents at higher risk of contracting an STI [37]. It is believed that the type of partner influences the STI risk of women because the prevalence is higher in single subjects [38,39]. However, researchers generally restrict the type of partner to labels of “casual” versus “fixed” or “living in union” versus “single”, which can mask the variability of risk [40].

Only 1% of the adolescents used condoms in all sexual reactions. Behavioral and social factors can lead adolescents to riskier sexual relationships, which favors constant exposure to and a higher risk of STIs [41]. These situations are closely related to the high morbidity of STIs in this population, and it is estimated that adolescents are responsible for approximately 50% of new STI cases each year, while approximately 24% of adolescents between 14 and 19 years of age have 1 out of 5 STIs commonly reported [5,9]. These infections may have important and irreversible health consequences for these girls and their unborn babies [5,17].

There were no records of patients with hepatitis B, hepatitis C, or HIV in our study. We believe that this extremely low prevalence was due to the flow of referrals of adolescents to the family planning outpatient clinic, as most of these patients were referred from a risk maternity unit where seropositive patients are not admitted for treatment because they are referred to reference maternity hospitals. In terms of viral hepatitis, since 1996, the National Immunization Program of the Ministry of Health of Brazil has recommended universal vaccination of children against hepatitis B at birth, i.e., all patients included in the study would have already had the vaccination and therefore should be immunized against hepatitis B [42].

The high frequency of STIs found in our study, especially in association with an absence of symptoms, is very worrisome. The low symptomatology can perpetuate the transmissibility of these diseases in a vulnerable group, with potential sequelae in the medium and long term. Clinical diagnosis based on symptoms of infections caused by *C*. *trachomatis* and *N*. *gonorrhoeae* has low sensitivity, and up to 70% of infected women may be asymptomatic [43]. Therefore, the importance of this study from an epidemiological point of view is fundamental, as there has been no study on the prevalence of STIs in this part of the western Amazon. These results can serve as a basis for more effective public measures in the region.

Brazil is already planning to implement measures to track STIs. For example, the Ministry of Health recommends annual screening for HIV and syphilis for adolescents and sexually active adolescents up to 30 years of age and for pregnant women in the first prenatal visit, beginning of the 3rd trimester and at the time of delivery [44]. In addition, chlamydia and gonococcus screening is recommended for the general population according to risk and for all pregnant women under 30 years of age [44]. Despite those recommendations, considering the data from Brazilian studies on the prevalence of STIs, we can say that the implementation of these recommendations is far from realized. If these STIs are not diagnosed and treated, they can lead to important obstetric and gynecological complications that considerably increase the costs of treatment and hospitalization and negatively impact female morbidity rates, especially in adolescents [45].

The high prevalence detected in our study, which is higher than the average rate for the rest of Brazil, is astonishing. Although our study was conducted in only one family planning outpatient clinic with a specific group of patients—girls between 14 and 19 years old—the findings demonstrate that improving control measures is crucial to prevent STIs among adolescents. Reducing the prevalence of STIs requires the implementation of public policies that disseminate knowledge about STIs and their forms of transmission and prevention, including consistent condom use, vaccination, improved screening, appropriate diagnosis, and timely treatment according to the best practices recommended by the WHO.

Although we performed the sample size calculation before the study, and we evaluated a specific group, adolescent girls, one of the limitations of this study may be related to the sample size. The sample size may be relatively small compared to the vast universe of communities in the Brazilian Amazon region, which have different difficulties in accessing social and health care, together with possible social biases in the adolescents’ responses to the questionnaire. In the context of these considerations, this preliminary study is part of a larger project that aims to identify potential indicators that will support more effective health care strategies aimed at improving the quality of life of populations in this region of the western Amazon. Although it should be possible to extrapolate these findings to other similar Amazonian communities, we must keep in mind that more research will be important, especially with other groups, to elucidate the prevalence of STIs and, therefore, use these data to improve public policies.

## Conclusion

The prevalence of STI among adolescents treated at the family planning outpatient clinic in the western Amazon region of Brazil is quite high, with *Chlamydia trachomatis* being the most common single STI found in this population. Our results reinforce the importance of expanding epidemiological research in the western Amazon and implementing more effective public measures toward the prevention of STIs among adolescents.
